# Identification of major cell types in paraffin sections of bovine tissues

**DOI:** 10.1186/1746-6148-2-5

**Published:** 2006-01-27

**Authors:** Mikael Niku, Anna Ekman, Tiina Pessa-Morikawa, Antti Iivanainen

**Affiliations:** 1 Department of Basic Veterinary Sciences, University of Helsinki, Helsinki, Finland

## Abstract

**Background:**

Identification of cell types in bovine tissue sections is complicated by the limited availability of anti-bovine antibodies, and by antigen retrieval treatments required for formalin-fixed tissue samples. We have evaluated an antibody and lectin panel for identifying major cell types in paraffin-embedded bovine tissue sections, and report optimized pretreatments for these markers.

**Results:**

We selected 31 useful antibodies and lectins which can be used to identify cell types of epithelia, connective tissue, muscle, and nervous tissue, as well as cell proliferation and apoptosis.

**Conclusion:**

The panel of markers allows the identification of all major cell types in paraffin-embedded cattle tissue sections by immunohistochemistry or lectin histochemistry. Heat-induced epitope retrieval methods are required for most antibodies.

## Background

Specific identification of cell types in bovine tissues is hindered by the limited availability of anti-bovine antibodies. The species cross-reactivity information of other commercially available antibodies is also often limited. Thus, suitable antibodies must be searched for by trial and error. This is further complicated by the fact that for many antibodies a successful immunostaining is only accomplished after an optimized antigen retrieval treatment.

As a by-product of a research project on stem cell fates[1], we have evaluated a selection of antibodies for identifying major bovine cell types in paraffin-embedded tissue sections. Some of these have been raised against bovine antigens, some are previously known to be bovine cross-reactive, and others we have tested without such prior knowledge. Optimal antigen retrieval methods for each antibody are reported. In addition to antibodies, two lectins are presented. The emphasis is on paraformaldehyde-fixed tissues, but as some antibodies are incompatible with such material, we have also used ethanol fixation.

## Results and discussion

**Figure 1:**
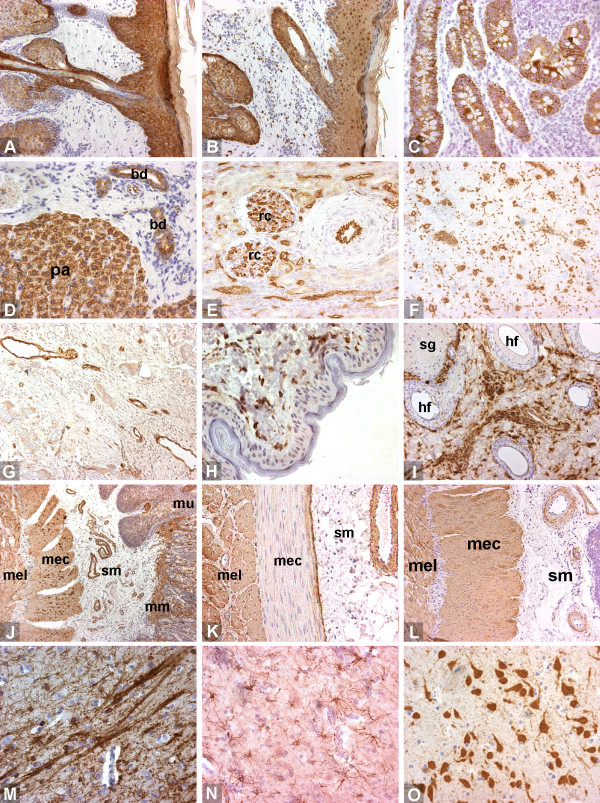
**Staining examples 1/2**. A. cytokeratin HMW, epidermis. B. cytokeratin, pan (polyclonal), epidermis. C. keratin, pan (AE1/AE3), intestinal epithelium. D. keratin, pan (Lu-5), liver. E. lectin GSL I-B4, kidney. F. lectin ML-I, brain. G. von Willebrand, granulation. H. procollagen type I, skin. I. vimentin, skin. J. actin (muscle), intestine. K. actin (smooth muscle α), intestine. L. desmin, intestine. M. CNPase, brain. N. GFAP, brain. O. NeuN, brain. pa: parenchyma, bd: bile duct, rc: renal corpuscle, sg: sebaceous gland, hf: hair follicle, mel: muscularis externa (longitudinal layer), mec: muscularis externa (circular layer), sm: submucosa, mm: muscularis mucosae, mu: mucosa.

**Figure 2:**
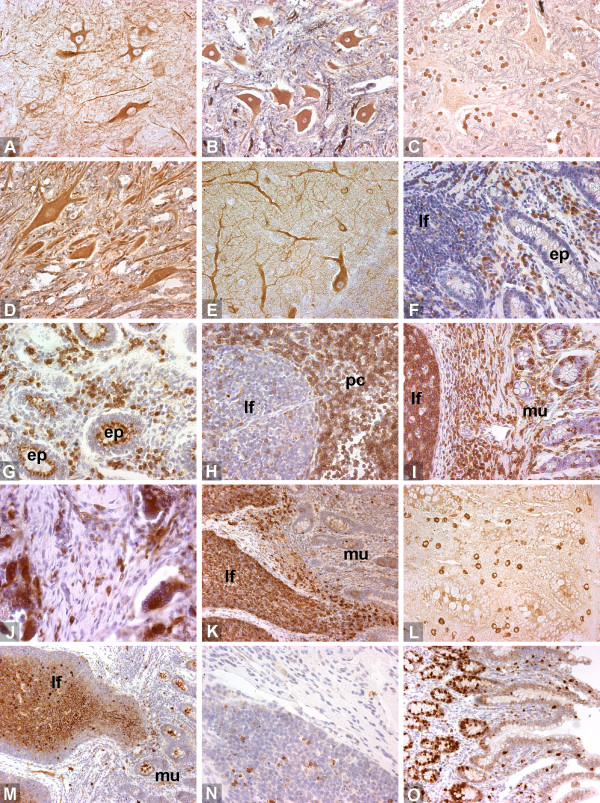
**Staining examples 2/2**. A. neurofilament (160/200 kD), brain. B. neurofilament (pan), spinal cord. C. S100, brain. D. tubulin βIII (polyclonal), spinal cord. E. tubulin βIII (TU-20), brain. F. CD11a/18 (BAT75A), intestinal mucosa. G. CD11b, intestinal mucosa. H. CD3ε, lymph node. I. CD45 (CACTB51A), intestine. J. CD68, granulation. K. CD79αcy, intestine. L. IgA, intestinal mucosa. M. IgM (Big73A), intestine. N. cleaved caspase 3, intestinal lymphoid follicle. O. Ki-67 antigen, intestinal mucosa. lf = lymphoid follicle, ep = epithelium, pc = paracortical area, mu = mucosa.

The marker panel is summarized in table I, with results from various antigen retrieval treatments tested. Succesfully stained tissue sections are presented in figures 1 and 2. The results are further commented below, including notes on any nonspecific staining detected.

**Table 1:** Evaluation of markers. ++: good staining (strong, specific), +: poor staining (weak or including non-specifically stained cells), -: unsuccesful staining. PFA: paraformaldehyde fixation, EtOH: ethanol fixation. N: no epitope retrieval, P: protease-induced epitope retrieval, H3: heat-induced epitope retrieval (HIER) at pH 3, H6: HIER at pH 6, H9: HIER at pH 9.5. Methods: A = avidin-biotin-complex, T = tyramide signal amplification. NF = neurofilament. Sources: DHSB = Developmental Studies Hybridoma Bank, BD = BD Biosciences, N/L = NeoMarkers/LabVision, Vector = Vector Laboratories, W/H = Witten/Herdecke University, Ch = Chemicon, SM = Sternberger Monoclonals, b/C = bAbco/Covance, VMRD: Veterinary Medicine Research Diagnostics, SC = Santa Cruz, BL = Bethyl Laboratories, Bm = Biomeda, CST = Cell Signaling Technology.

**Marker**	**clone/type**	**Immunogen**	**Source**	**Ref.**	**Staining**							**Dilution (method)**
						
					**PFA**					**EtOH**		
						
					**N**	**P**	**H3**	**H6**	**H9**	**N**	**P**	
EPITHELIUM, ENDOTHELIUM												
collagenType IV	M3F7	human collagen	DSHB	[10]	-	-	-	-	-	-	-	1:200T
cytokeratin, HMW	34betaE12	human epid. keratin	Dako	[5]				++	-	++		1:1000T
cytokeratin, pan	rabbit polycl.	bovine epid. keratin	Zymed	n.a.	+	+		+		+		1:1000T
eNos/NOSType III	rabbit polycl.	peptide (human)	BD	[11]			+	-	+	+		1:1000T
keratin, pan	AE1 + AE3	human epid. keratin	N/L	[2,3]	-	++	-	++	++	++		1:100A, 1:1000T
keratin, pan	Lu-5	lung cancer cell line	N/L	[4]		++		++	-	++		1:100A, 1:1000T
lectin GSL I-B4	*G. simplicifolia*	n.a.	Vector	[8,9]	++	++		++		++		1:4000T
lectin ML-I	*Viscum album*	n.a.	W/H	[7]	++	++	++	++	++			1:2000T
von Willebrand	rabbit polycl.	human vWF	Dako	[6]	+						++	1:400A, 1:800T
CONNECTIVE TISSUE												
procollagenType I	SP1.D8	ovine aminopropept.	DSHB	[14]	+	+	+	+	++	-	-	1:2000T
vimentin	V9	porcine vimentin	Dako	[12,13]	++			++			++	1:100A, 1:500T
MUSCLE												
actin, muscle	HHF35	n.a.	Enzo	[17]	+		++	+	++	++	++	1:100A
actin, smooth muscle α	1A4	peptide	Dako	[18]				++	+			1:500T
desmin	D33	human desmin	Dako	[15,16]	+			++		++		1:400A, 1:2000T
NEURONAL TISSUE												
CNPase	11-5B	human brain CNPase	Ch	n.a.		++	-	++	-	-		1:400A
GFAP	rabbit polycl.	human GFAP	Zymed	n.a.	++	++	+	++	++	++		1:200A
NeuN	A60	mouse neuronal nuclei	Ch	[19,22]	+	+	-	+	++	++	+	1:4000T
NF 160/200 kD	RMdO-20	rat neurofilaments	Zymed	[22]	++		-	-	++	++		1:400A
NF, pan	SMI311 (cockt)	n.a.	SM	[21]	++		+	++	++	+		1:1000A, 1:2000T
O4	81	bovine brain	Roche	[23]			-	-	-		-	1:2A
S100	rabbit polycl.	bovine S100	Dako	[24]				++				1:800A
tubulin βIII	rabbit polycl.	peptide (rat)	b/C	n.a.			-	++	-	+		1:6000A,1:10000T
tubulin βIII	TU-20	n.a.	Ch	[20]	++		++	++	++	++		1:400A, 1:3000T
LEUKOCYTES												
CD11a/18	MUC76A	sheep, pig leukocytes	VMRD	[28]	-		-	-	-			1:40A
CD11a/18	BAT75A	ruminant leukocytes	VMRD	[27]	-		++	-	-	+		1:40A, 1:200T
CD11b	MM10A	n.a.	VMRD	[32]			++			++		1:500T
CD14	MM61A	bovine mononucl. cells	VMRD	[25]	+					+		1:200
CD34	rabbit polycl.	peptide (human)	SC	n.a.			+	-	-			1:200A, 1:200T
CD3ε	rabbit polycl.	peptide (human)	Dako	[29]	-			++		+	++	1:100A, 1:1000T
CD45	CACTB51A	bovine act. lymphocytes	VMRD	[25]		+	+	+	+	+	++	1:800T
CD45	CC1	n.a.	Serotec	[26]				+	+			1:100A
CD68	EBM11	human macrophages	Dako	[33]	-	+		-			++	1:80A
CD79αcy	HM57	peptide (human)	Dako	[30]			-	+	++			1:500T
IgA	rabbit polycl.	bovine IgA	BL	n.a.		++			++		++	1:100A
IgM	rabbit polycl.	bovine IgM	BL	n.a.	+				++			1:1500A
IgM	BIg73A	bovine Ig	VMRD	[31]			-	+	++			1:5000A
lysozyme	rabbit polycl.	human lysozyme	Bm	[34]		+	-	-	+	+	+	1:500T
CELL STATUS												
cleaved caspase 3 (Asp175)	rabbit polycl.	peptide (human)	CST	[37]				++	+			1:75A
Ki67 antigen	MIB-1	peptide (human)	Dako	[35,36]				-	++			1:4000T

### Epithelium

Several anti-keratin antibodies were evaluated for epithelial markers. The AE1/AE3 monoclonal antibody cocktail[2,3], raised against human epidermal keratin, was the most useful pan-epithelial marker. With alkaline antigen retrieval (see Methods), most types of epithelia were strongly and specifically stained with this antibody. Neutral or protease-induced retrieval was sufficient for some but not all tissues.

Another pan-keratin antibody Lu-5[4] also stained most epithelia with protease-induced retrieval, but did not cover all epithelia as comprehensively as AE1/AE3 in our hands.

The polyclonal pan-keratin antibody tested yielded nonspecific staining of cells. The high molecular weight cytokeratin antibody 34β E1[5] provided very strong staining of some epithelia (notably epidermis and liver), but is obviously not as comprehensive as the pan-keratin markers.

Few bovine endothelial markers are available. The anti- von Willebrand antibody[6] tested stains many but not all endothelia. Notably, new blood vessels in granulation tissue were strongly stained. The lectins ML-I[7] and especially GSL I-B4[8,9] yielded strong staining of endothelial cells, but they also stain some leukocyte populations. We failed to obtain a good staining with the anti- type IV collagen antibody M3F7[10] or with the polyclonal anti- endothelial nitric oxide synthase antibody[11].

### Connective tissue

Vimentin is a general marker for cells of the mesenchymal lineage. The monoclonal anti- porcine vimentin antibody V9[12,13] provided strong and specific staining even without antigen retrieval.

The type I procollagen antibody SP1.D8 [14] stained active fibroblasts in various connective tissues. The best staining was obtained with the alkaline retrieval method. Some nonspecific staining was seen, in sebaceous glands for example.

### Muscle

The desmin antibody D33 stained all muscle types, performing best after neutral antigen retrieval [15,16]. The muscle actin antibody HHF35 [17] recognizes the alpha and gamma isotypes present in all muscle types. The acid and alkaline retrieval methods yielded optimal results. The α-smooth muscle actin antibody 1A4 [18] stains only a subset of smooth muscle tissues, due to the more restricted expression pattern of the antigen. The circular muscle layer in the intestine was not stained. The neutral antigen retrieval method produced the best results with this antibody. All these antibodies were specific for muscle tissues.

### Nervous tissue

The monoclonal anti- NeuN antibody A60 [19] was the most comprehensive neuronal marker tested. It stained most neurons, Purkinje cells being a notable exception. Best results were obtained with alkaline retrieval.

Tubulin and neurofilament antibodies are also useful as general neuronal markers. Majority of neurons were stained by both tubulin βIII antibodies [20] and the pan-neurofilament cocktail[21], and more restricted subgroups by the neurofilament 160/200 kD antibody[22], as expected.

Astroglia were beautifully stained with the polyclonal anti-GFAP antibody, with most pretreatments. Oligodendroglia were successfully stained with the anti-CNPase antibody 11-5B, whereas the anti-O4 antibody 81[23] failed to produce any specific staining in our hands. S100[24] is a more general marker for glial cells. It is also expressed in several cell types outside the nervous system.

The microglial cells were very weakly stained with the pan-leukocyte and macrophage markers tested. The mistletoe lectin ML-I[7] yielded successful staining with all pre-treatments. It also stains most endothelial cells.

### Leukocytes

Leukocyte markers are often species-specific, and most commonly used in flow cytometry. Thus, most of the antibodies tested were raised against bovine antigens, but information on histological applications was limited.

Of the pan-leukocyte markers, the anti- CD45 antibody CACTB51A[25] worked well on ethanol-fixed material with mild protease treatment, except for the microglia, which were weakly stained. The quality of the CC1 antibody[26] appeared to suffer from a change in the production method during the research project.

We tested two CD11a/18 antibodies, of which BAT75A[27] was useful with acid antigen retrieval, while MUC76A[28] failed with any pre-treatment.

For lymphocytes, the CD3ε (T cells) and CD79α (B cells) antibodies [29,30] raised against synthetic cytoplasmic peptides worked well with antigen retrieval, providing a strong and specific staining. The anti- immunoglobulin antibodies successfully stained B cells with alkaline retrieval[31].

Myeloid cells were stained with the anti- CD11b antibody MM10A[32] using acid retrieval or ethanol-fixed material. The macrophage marker CD68[33] was also useful with protease-treated sections, but did not stain microglia. The CD14 antibody MM61A[25] was only useful with ethanol-fixed material. The polyclonal anti-lysozyme antibody[34] stained a number of cells in the intestinal epithelium, for example, but we failed to confirm the specificity of the staining.

### Cell status

The proliferation marker MIB-1[35,36] yielded good staining with alkaline antigen retrieval. Apoptotic cells were successfully stained with the cleaved caspase 3 antibody[37] using neutral retrieval, although the staining was not very strong.

## Conclusion

In order to facilitate identification of major cell types in paraffin sections of bovine tissues, we evaluated a number of markers for immunohistochemistry and lectin histochemistry. Antibodies raised against bovine antigens or known to be bovine cross-reactive were used where possible. If no information on bovine reactivity was available, antibodies with broadest species cross-reactivity were selected. Two lectins were used in addition to the antibody markers.

The panel of 31 useful markers allows the identification of all major cell types in cattle tissue sections. With paraformaldehyde-fixed material, heat-induced antigen retrieval is beneficial for most antibodies. By selecting a suitable retrieval protocol, most markers can be successfully applied to this type of material.

## Methods

### Tissue samples

Tissue samples were obtained from slaughtered animals. The use of animals was approved by the animal ethics committee of the University of Helsinki. Tissue samples were fixed either in 4% phosphate-buffered paraformaldehyde (PFA) for 24 hours at +4°C or in 100 % ethanol for 2 hours at +4°C followed by 120 hours at -20°C, embedded in paraffin, and sectioned to 2–4 μm (PFA) or 4 μm (ethanol) sections.

### Antibodies and lectins

All reagents are commercially available. The sources of antibodies and lectins are listed in table 1.

### Immunohistochemistry

Immunohistochemistry was performed using either the ABC method (avidin biotin complex) or tyramide amplification, using Shandon Coverplates (ThermoElectron). Paraffin-embedded sections were dewaxed, rehydrated, subjected to an antigen retrieval procedure (see below), and permeabilized with 0.1% to 1% Tween-20 in phosphate-buffered saline (PBS). The sections were then blocked for endogenous biotin, with 10% egg white powder in water (as an avidin solution) and 1 mg/ml D-biotin (Sigma-Aldrich, St. Louis, MO) in PBS, when necessary, and for nonspecific binding with 1% goat serum in PBS. They were incubated in the primary antibody overnight at +4°C, in PBS containing 1% bovine serum albumin, washed, and incubated with goat biotinylated anti-mouse or anti-rabbit secondary antibody (Dako, Glostrup, Denmark) for 2 hours in room temperature. The ABC detection was performed using the Vectastain Elite ABC kit and the DAB (diaminobenzidine) substrate kit (both Vector Laboratories, Burlingame, CA) according to manufacturer's instructions. For tyramide amplification, sections were incubated in avidin D -conjugated peroxidase (Vector), in biotinylated tyramide [38], again in avidin-peroxidase, and in the DAB substrate. The amplification typically allows four to ten times more dilute antibody solutions than with the ABC method. All sections were counterstained with Mayer's hematoxylin and embedded with Faramount (Dako).

### Antigen retrieval

Heat-induced antigen retrieval was performed in a standard kitchen microwave oven. The slides were heated in 500 ml of retrieval solution at 750 W power for 15 minutes (for the caspase antibody, 10 minutes), followed by a cooling period of 20 minutes (for the caspase antibody, 30 minutes). The following solutions were used: for acid retrieval, 50 mM glycine-HCl pH 3; for neutral retrieval, 2 × SSC pH 6 (sodium chloride, sodium citrate buffer); and for alkaline retrieval, 10 mM Tris-HCl pH 9.5, 1 mM EDTA.

Protease-induced antigen retrieval was performed in Coverplates, at 37°C for 30 minutes, with 10 to 50 μg/ml (ethanol-fixed samples) or 50 to 100 μg/ml (PFA-fixed samples) protease P6911 (Sigma-Adrich) in 10 mM Tris-HCl pH 7.4, 0.5 mM EDTA.

### Microscopy and photography

The stained sections were viewed with a Leica DM4000 microscope and photographed using a SIS Colorview 12 digital camera.

## Authors' contributions

MN designed the staining tests and optimized the pretreatments, selected most markers, wrote the manuscript and prepared the figures. AE and TPM participated in the selection of markers and in the staining tests. AI participated in the selection of markers and in the preparation of the manuscript. All authors have approved the final manuscript.
